# A Cross-Sectional Study of Endometriosis Surgeries Across Obstetrics and Gynecology Subspecialties: Does Minimally Invasive Gynecologic Surgery (MIGS) Fellowship Training Make a Difference?

**DOI:** 10.7759/cureus.90828

**Published:** 2025-08-23

**Authors:** Soha Wahab, Zeina Chehade, Miran Jaffa

**Affiliations:** 1 Faculty of Medicine, American University of Beirut, Beirut, LBN; 2 Obstetrics and Gynecology, University of Texas Health Science Center at San Antonio, San Antonio, USA; 3 Epidemiology and Biostatistics, American University of Beirut, Beirut, LBN

**Keywords:** deep endometriosis, deep endometriosis surgery, deep infiltrative endometriosis, endometriosis and chronic pelvic pain, endometriosis excision, laparoscopic surgery for endometriosis

## Abstract

Objective

The objective of this study was to compare the surgical approaches and postoperative outcomes of endometriosis surgeries performed by different obstetrics and gynecology (OBGYN) subspecialties: minimally invasive gynecologic surgery (MIGS), gynecologic oncology (GONC), and other OBGYN subspecialties (OBGYNS).

Methods

This was a retrospective cross-sectional study on patients who presented to a tertiary healthcare center between 2018 and 2019 and had undergone their first endometriosis surgery at the same center in 2000 or later (n = 258). Variables were compared across subspecialties using Chi-squared and Fisher’s exact tests. A multinomial multivariable logistic model (MMLM) was applied to assess adjusted associations between subspecialty, presence of deep endometriosis (DE), and surgical techniques used.

Results

Univariate analysis revealed significant differences among subspecialties in several areas (p < .001). First, laparoscopy was predominantly used by MIGS (99.3%) and OGYNS (87.9%), while laparotomy was common in GONC cases (66.7%). Pain was the primary indication for MIGS (92.9%), whereas 45% of GONC cases had other indications, like large endometriomas or postmenopausal cysts. OGYNS favored ablation (77.6%), including 85.7% of DE cases. MIGS employed excision in 30.7% of patients and a combination of excision and ablation in 33.6% of cases, with excision not necessarily complete. GONC primarily used ablation (48.3%) and performed isolated endometrioma removal (38.3%). DE was excised in 34.4% (MIGS), 18.8% (GONC), and 0% (OGYNS) of DE cases. Postoperative pain outcomes did not differ significantly (p = .735); persistent pain was reported in MIGS (76.9%), GONC (78.1%), and OGYNS (84.4%). MMLM indicated GONC (p = .011and p = .006) and OGYNS (p = .001 and p = .017) were significantly more likely than MIGS to perform ablation or remove endometriomas alone when adjusting for DE presence.

Conclusion

Despite variations in surgical techniques among subspecialties, persistent postoperative pain and low DE excision rates underscore the need for standardized endometriosis training and specialized referrals.

## Introduction

Endometriosis is a systemic inflammatory disease that affects more than 190 million individuals worldwide [1]. It is characterized by the presence of endometrial-like tissue outside the uterus, most commonly within the pelvic and abdominal cavities [2]. Clinical manifestations are heterogeneous and may include chronic pelvic pain, infertility, and a range of other symptoms that can significantly impair quality of life. Currently, no curative medical therapy exists. Available pharmacologic treatments are primarily focused on symptom management, particularly pain relief, and there is a lack of high-level evidence supporting the superiority of one therapeutic option over another [3]. Surgical excision remains the gold-standard therapeutic approach, with optimal outcomes observed when procedures are performed in specialized centers [4,5]. Such specialization is associated with lower complication and recurrence rates, as well as significant improvements in both pain and fertility following surgery [4-6].

Despite growing evidence supporting the benefits of specialized care, particularly in the surgical management of endometriosis, there is no established consensus on the standardized components of multidisciplinary specialized care. Moreover, there is currently no formally accredited fellowship program dedicated exclusively to the comprehensive management of endometriosis, despite the disease’s complexity and data indicating that higher surgical volumes and greater surgeon experience are associated with improved outcomes [7-9].

The primary objective of this study is to evaluate surgical characteristics, techniques, and postoperative outcomes of endometriosis surgeries performed by different obstetrics and gynecology (OBGYN) subspecialties, including minimally invasive gynecologic surgery (MIGS), gynecologic oncology (GONC), and other OBGYN subspecialties (OBGYNS). MIGS and GONC are the two OBGYN subspecialties that most frequently operate on complex endometriosis cases; therefore, they were analyzed as separate categories. Such a comparison would provide valuable insights into the impact of specialization on surgical outcomes in non-specialized settings and underscore the need for establishing standardized surgical training and competency benchmarks. Data were collected from a tertiary care center that lacks specialized endometriosis services, reflecting the structure of many institutions worldwide that continue to manage a substantial proportion of patients with this condition. To date, no studies have directly compared outcomes across OBGYN subspecialties in the context of endometriosis surgery. Such real-world data analysis is essential to inform future strategies for optimizing the care and surgical management of patients with endometriosis.

## Materials and methods

This was a retrospective cross-sectional study employing convenience sampling conducted at American University of Beirut Medical Center, Beirut, Lebanon. The study was approved by the Institutional Review Board of the American University of Beirut Medical Center (BIO-2021-0372, dated January 17, 2022). All patient information was de-identified, and patient consent was not required.

Eligibility criteria

Patients aged 18 and older who presented to a tertiary healthcare center between 2018 and 2019, with medical records indicating that their first endometriosis surgery at the institution took place in or after the year 2000 (N = 258) were included.

Quality measures

Data collection and analysis were conducted following approval by the center’s Institutional Review Board (IRB) and in accordance with ethical research guidelines. Clinical data were extracted from the EPIC electronic medical record (EMR) system (Epic Systems Corporation, Verona, Wisconsin, United States), which was implemented in 2018. All data were de-identified prior to analysis, and no patients were contacted as part of this study. One author verified the collected data by cross-referencing EMR records, and discrepancies were resolved by consensus.

Data collection

Several surgical and postoperative variables were evaluated, including the type of surgery, intraoperative findings, surgical techniques used to remove endometriosis, and postoperative pain outcomes. Surgical techniques were classified based on operative reports as: (i) excision (i.e., resection of endometriosis or excision of lesions) and (ii) ablation (i.e., lesions cauterized or ablated). Additionally, we added a third category for cases in which no peritoneal lesions were treated in the presence of endometriomas. Due to the absence of a standardized pain scoring system in patient charts, pain outcomes assessed six weeks after surgery were categorized as either "persistent" or "improving" based on clinical follow-up notes compared with preoperative documentation. A small subset of patients lacked any documentation regarding postoperative pain status.

Surgical subspecialties were defined by the type of fellowship training completed after OBGYN residency, either in the United States or an equivalent European system. Infertility was defined as the inability to conceive after one year of unprotected intercourse in individuals under 35, or after six months in those aged 35 and older. The presence of deep endometriosis (DE) was determined based on operative reports and surgical notes, using the following criteria: partial or complete obliteration of the cul-de-sac, frozen pelvis, stage 3 or 4 endometriosis, mention of severe disease, or the identification of a characteristic DE lesion (e.g., fibrotic nodule in the bowel wall). If DE findings were not clearly documented as present or absent, intraoperative DE status was recorded as missing.

Statistical analysis

General characteristics of the patient population were summarized using means, standard deviations (SD), medians, and ranges for continuous variables, and frequencies with percentages for categorical variables. Variables were compared across subspecialties using Chi-squared and Fisher’s exact tests Tables 2-4. In Table 5, a multinomial multivariable logistic model (MMLM) was applied to assess adjusted associations between surgical subspecialty, the presence of DE, and the surgical techniques employed. A significance level of ≤ 0.05 was used for all statistical tests. Analyses were conducted using Stata Statistical Software: Release 17 (StataCorp LLC., College Station, Texas, United States).

## Results

A total of 258 patients were included in the study. Table 1 presents the general characteristics of the study population. The mean follow-up duration was 6.4 years, and the mean age at the time of the first endometriosis surgery was 32 years. On average, patients underwent 1.17 surgeries during the follow-up period, with a range of one to five surgeries. The average minimum interval between two consecutive surgeries was approximately 3.5 years, ranging from six months to 14 years. Only 112 (43.4%) patients underwent pelvic MRI within six months of the first endometriosis surgery, which is the only imaging modality available for the detection of DE. Among those who had an MRI, 64 (57.1%) had findings suggestive of DE.

**Table 1:** General characteristics of patients undergoing first-time endometriosis surgery in a non-specialized healthcare system (2000–2023) (N=258) Numerical variables represented as frequency (percentage); Continuous variables represented as mean (SD) and median (range) DE: deep endometriosis

Characteristics	Frequency (Percentage)	Mean	Standard Deviation	Median	Range
Number of surgeries done per patient	-	1.17	0.5	1	(1-5)
Age at first surgery	-	32	7.1	31.5	(19-51)
Minimum number of months between two endometriosis surgeries	-	44.3	45.3	36.4	(6-168)
MRI done within 6 months of the first endometriosis surgery	112 (43.4)	NA	NA	NA	NA
DE diagnosed on MRI	64 (57.1)	NA	NA	NA	NA
Length of follow-up (years)	-	6.4	5.3	5	(1-28)

Table 2 highlights significant differences in surgical characteristics among endometriosis cases managed by different OBGYN subspecialties. MIGS performed the highest proportion of minimally invasive surgeries of 139 (99.3%) compared to 20 (33.3%) for GONC and 51 (87.9%) for OGYNS (p < .001). Indications for surgery also varied significantly across subspecialties. MIGS cases were predominantly performed for pain (n=119; 85%), whereas GONC cases showed a more heterogeneous distribution, with 27 (45%) surgeries indicated for factors such as large endometriomas, history of cancer, as well as abnormal uterine bleeding or ovarian cysts in postmenopausal patients (p < .001). MIGS were more likely to perform excision (n=43; 30.7%) or combined excision and ablation (n=47; 33.6%), although excision was not necessarily complete. In contrast, ablation alone was most common among GONC (n=29; 48.3%) and OGYNS (n=45; 77.6%) (p < .001). Additionally, DE was more frequently diagnosed intraoperatively in MIGS cases (n=93; 66.4%) compared to GONC (n=32; 53.3%) and OGYNS (n=28; 48.3%) (p = .002). Among patients with DE, MIGS were significantly more likely to perform combined excision and ablation (n=41; 44.1%), while ablation alone dominated in surgeries by GONC (n=22; 68.8%) and OGYNS (n=24; 85.7%) (p < .001).

**Table 2:** Comparison of endometriosis surgical characteristics across MIGS, GONC, and OBGYNS (2000–2023) (N=258) ^b^ Others: large endometrioma, history of cancer, abnormal uterine bleeding, cyst in a post-menopausal patient Variables represented as n (%); Variable categories were compared using the Chi-Squared Test; Significance: p < 0.05 MIGS: minimally invasive gynecologic surgery; DE: deep endometriosis; OBGYNS: other gynecological subspecialties; GONC: gynecologic oncology

Variable	Category	Endometriosis Surgeries by MIGS (n=140), n (%)	Endometriosis Surgeries by GONC (n=60), n (%)	Endometriosis Surgeries by OBGYNS (n=58), n (%)	Statistic Value	P-value
Type of Surgery	Laparotomy	1 (0.7)	40 (66.7)	7 (12.1)	122.75	< 0.001
	Minimally invasive	139 (99.3)	20 (33.3)	51 (87.9)		
Indication for the Surgery	Pain Only	119 (85)	30 (50)	30 (51.7)	84.66	< 0.001
	Infertility Only	5 (3.6)	1 (1.7)	7 (12.1)		
	Pain & Infertility	11 (7.9)	2 (3.3)	15 (25.9)		
	Others ^b^	5 (3.5)	27 (45)	6 (10.3)		
Surgical Technique	Excision Only	43 (30.7)	7 (11.7)	1 (1.7)	101.057	< 0.001
	Ablation Only	35 (25)	29 (48.3)	45 (77.6)		
	Excision & Ablation	47 (33.6)	1 (1.7)	0 (0)		
	Only endometrioma was removed/drained	15 (10.7)	23 (38.3)	12 (20.7)		
DE diagnosed in Surgery	Yes	93 (66.4)	32 (53.3)	28 (48.3)	12.141	0.002
	No	12 (8.6)	12 (20)	15 (25.9)		
	Missing	35 (25)	16 (26.7)	15 (25.8)		
Surgical Technique in Patients with DE	-	N = 93	N = 32	N = 28	57.434	< 0.001
	Excision Only	26 (28)	5 (15.6)	1 (3.6)		
	Ablation Only	23 (24.7)	22 (68.8)	24 (85.7)		
	Excision & Ablation	41 (44.1)	1 (3.1)	0 (0)		
	Only endometrioma was removed/drained	3 (3.2)	4 (12.5)	3 (10.7)		

As shown in Table 3, the type of endometriosis excised varied significantly across subspecialties (p < .001). Endometriomas were the most commonly excised lesions in all groups, with the highest proportion in GONC (52; 86.7%). DE was the least frequently excised overall, though it was significantly more likely to be excised by MIGS surgeons (n=32; 22.9%) compared to GONC (n=6; 10%) and was never excised by OGYNS (n=0; 0%). Additionally, 17 (29.3%) patients treated by OGYNS had no endometriotic tissue excised, compared to only six (4.3%) in the MIGS group and three (5%) in the GONC group. When DE was excised, it was most commonly excised from the posterior compartment or uterosacral ligaments, both in surgeries performed by MIGS (n=28; 87.5%) and GONC (n=3; 50%). Bowel and bladder DE excision was rare, while diaphragmatic lesions were never excised by any group. Endometrioma management did not significantly differ across groups (p = .064), with excision being the predominant approach over oophorectomy across all subspecialities. Notably, alternative techniques such as sclerotherapy and plasma energy ablation were not used in any case. Specimens other than endometriomas were sent to pathology in 87 (62.1%) MIGS cases, compared to only seven (11.7%) and one (1.7%) in the GONC and OGYNS groups, respectively (p < .001). Among these, most showed histopathologic confirmation of endometriosis, with a minority revealing only fibrosis, particularly in the MIGS (n=8; 5.7%) and GONC (n=1; 14.3%) groups.

**Table 3:** Comparison of endometriosis surgical management by MIGS, GONC, and OBGYN (2000–2023) (N=258) ^d^ Fisher’s Exact Variables represented as n (%); Variable categories were compared using Chi-Squared and Fisher's Exact Tests; Significance: p < 0.05 MIGS: minimally invasive gynecologic surgery; DE: deep endometriosis; USL: uterosacral ligament; GONC: gynecologic oncology; OBGYNS: other gynecologic subspecialties

Variable	Category	Endometriosis Surgeries by MIGS (n=140), n (%)	Endometriosis Surgeries by GONC (n=60), n (%)	Endometriosis Surgeries by OBGYNS (n=58), n (%)	Statistic Value	P-value
Type of endometriosis excised	DE	32 (22.9)	6 (10)	0 (0)	82.853	< 0.001
	Superficial	57 (40.7)	4 (6.7)	1 (1.7)		
	Endometrioma	95 (67.9)	52 (86.7)	40 (69)		
	Nothing was excised	6 (4.3)	3 (5)	17 (29.3)		
DE excised from	-	n = 32	n = 6	n = 0	6.863	0.054^d^
	Cul de sac or USL	28 (87.5)	3 (50)	--		
	Bowel	2 (6.3)	1 (16.7)	--		
	Bladder	1 (3.1)	1 (16.7)	--		
	Diaphragm	0 (0)	0 (0)	--		
	Parametrium	2 (6.3)	2 (33.3)	--		
Endometrioma management	-	n = 95	n = 52	n = 40	5.495	0.064
	Excised	88 (92.6)	42 (80.8)	37 (92.5)		
	Oophorectomy	7 (7.4)	10 (19.2)	3 (7.5)		
Cases in which specimens (other than endometrioma) were sent to pathology	Yes	87 (62.1)	7 (11.7)	1 (1.7)	85.618	< 0.001
	No	53 (37.9)	53 (88.3)	57 (98.3)		
Pathology results	-	n = 87	n = 7	n = 1	7.624	0.076^d^
	Endometriosis	70 (80.5)	3 (42.9)	1 (100)		
	Fibrosis	8 (5.7)	1 (14.3)	0 (0)		
	Endometriosis & Fibrosis	9 (6.4)	3 (42.9)	0(0)		

For patients who underwent endometriosis surgery for pain, Table 4 shows that postoperative pain outcomes did not significantly differ across subspecialties (p = .735), including those diagnosed intraoperatively with DE (p = .212). Persistent pain was reported in the majority of patients across all groups, with even higher rates observed among those with DE. However, it is important to note that postoperative pain assessment was based on physician documentation rather than standardized pain scoring tools. Regarding infertility outcomes, pregnancy was achieved in nine (56.3%) patients treated by MIGS, one (33.3%) by GONC, and 18 (81.8%) by OGYNS, though this difference did not reach statistical significance (p = .086).

**Table 4:** Postoperative outcomes of endometriosis surgeries performed by MIGS, GONC, and OBGYNS (2000–2023) (N=258) ^c^ Fisher’s Exact Variables represented as n (%); Variable categories were compared using Fisher's Exact Test; Significance: p < 0.05 MIGS: minimally invasive gynecologic surgery; DE: deep endometriosis; GONC: gynecologic oncology; OBGYNS: other gynecologic subspecialties

Variable	Category	Endometriosis Surgeries by MIGS (n=140), n (%)	Endometriosis Surgeries by GONC (n=60), n (%)	Endometriosis Surgeries by OBGYNS (n=58), n (%)	Statistic Value	p-value
Pain post-surgery	-	n = 130	n = 32	n = 45	2.059	0.735^c^
	Persistent	100 (76.9)	25 (78.1)	38 (84.4)		
	Improved	13 (10)	4 (12.5)	2 (4.4)		
	Cannot Tell	17 (13.1)	3 (9.4)	5 (11.1)		
Pain post-surgery in patients with DE	-	n = 87	n = 23	n = 26	5.361	0.212^c^
	Persistent	68 (78.2)	20 (87)	25 (96.2)		
	Improved	6 (6.9)	2 (8.7)	0 (0)		
	Cannot Tell	13 (14.9)	1 (4.3)	1 (3.8)		
Infertility	-	n = 16	n = 3	n = 22	4.638	0.086^c^
	Pregnancy achieved	9 (56.3)	1 (33.3)	18 (81.8)		
	Pregnancy not achieved	7 (43.8)	2 (66.7)	4 (18.2)		

Finally, Table 5 presents the results of MMLM examining associations between subspecialty, identification of DE during surgery, and the surgical technique used. This analysis revealed that GONC (p = .011 and p = .006) and OGYNS (p = .001 and p = .017) were significantly more likely than MIGS to perform ablation or remove endometriomas alone when adjusting for DE presence. On the other hand, isolated excision of endometrioma was associated with the absence of DE when controlling for the surgeon’s subspecialty (p < .001).

**Table 5:** Multinomial multivariable associations between subspecialty, presence of DE and surgical technique (2000-2023) (N = 258) Multinomial multivariable logistic model; Significance: p < 0.05 MIGS: minimally invasive gynecologic surgery; DE: deep endometriosis; GONC: gynecologic oncology; OBGYNS: other gynecologic subspecialties

Predictors	Categories	Ablation Only		Excision & Ablation		Only endometrioma was removed /drained	
		OR	p-value	OR	p-value	OR	p-value
Obgyn Subspecialty	MIGS (Ref)	NA	NA	NA	NA	NA	NA
	GONC	3.900	0.011	109	0.046	6.917	0.006
	OBGYNS	30.417	0.001	8.241x10^-7^	0.988	16.743	0.017
DE^b^ identified in surgery	Yes (Ref)	NA	NA	NA	NA	NA	NA
	No	3.158	0.161	3.143 x10^-7^	0.989	29.312	< 0.001

## Discussion

The most recent guideline on endometriosis management from the American College of Obstetricians and Gynecologists (ACOG) was released in 2010 [10]. Since then, substantial advancements have occurred in diagnostic approaches, surgical techniques, and classification systems [4-6,11-14]. A recent meta-analysis by Pundir et al. demonstrated that surgical excision of endometriosis, compared to ablation, significantly improves quality of life, as measured by the Endometriosis Health Profile-30 (EHP-30), and leads to meaningful reductions in multiple types of pain [13]. Furthermore, the surgical diagnosis of DE, particularly in locations such as the rectosigmoid and bladder, is less accurate than diagnosis using specialized imaging protocols, according to findings by Goncalves et al. [15]. Importantly, a multidisciplinary approach to endometriosis care that incorporates specialized imaging protocols to map DE lesions and coordinated surgical excision by a multidisciplinary team has been shown to significantly improve symptoms, including pain and infertility, while also reducing the likelihood of recurrent surgery for endometriosis [4-6,12-14].

Despite the complex nature of endometriosis presentation, diagnosis, and treatment, current guidelines do not specify the qualifications or expertise required of the treating team [10]. At present, the MIGS subspecialty remains the only accredited fellowship with substantial exposure to endometriosis, often receiving referrals for the most complex cases. Indeed, our data demonstrates that MIGS surgeons are more likely to perform laparoscopic surgery and use excision techniques compared to OBGYNS. However, in the MIGS category, only 32 of the 93 patients with intraoperatively diagnosed DE (33.4%) underwent excision of DE lesions, which was not necessarily complete, and the majority continued to experience persistent postoperative pain. Although pain assessment in our study was based on clinical documentation rather than standardized scoring systems, these outcomes are markedly different from those reported in surgeries performed at specialized endometriosis centers [5].

A population-based cohort study in Ontario, Canada, comparing endometriosis surgeries performed by surgeons with varying surgical volumes found that high-volume surgeons had lower rates of complications and repeat surgeries [8]. In fellowship programs accredited by the American Association of Gynecologic Laparoscopists (AAGL), there is no minimum requirement for the number of endometriosis surgeries performed. Such a requirement exists only for certification by the American Board of Obstetricians and Gynecologists (ABOG) in MIGS, which mandates a minimum of 100 minimally invasive procedures across various categories after graduation [16-18]. For GONC and for general OBGYN practitioners, no such surgical minimum exists. The absence of standardized requirements and evaluation for endometriosis surgical skills within MIGS fellowship programs contributes to variability in surgical expertise among MIGS-trained surgeons.

In recent years, endometriosis surgery has become more complex yet safer when performed in a specialized setting, largely due to innovations such as nerve-sparing techniques and the advancement of neuropelveology [19,20]. Despite these surgical improvements, training and competency standards for surgeons and medical teams managing endometriosis have not evolved accordingly. Many healthcare professionals, including MIGS surgeons, who lack sufficient exposure to specialized endometriosis care during their training, are often required to develop these skills independently. To demonstrate expertise in this complex field, an increasing number of surgeons and healthcare facilities seek external accreditation, such as that offered by the Surgical Review Corporation (SRC) [21,22]. This accreditation requires meeting specific criteria, such as performing a minimum number of endometriosis surgeries each year, managing complex cases, and providing patient education [21,22].

Another hurdle that surgeons aiming to provide specialized care face is a reimbursement structure that often fails to reflect the time, technical skill, and expertise required for managing complex endometriosis cases. This issue is not unique to endometriosis. A recent large-scale analysis highlighted systemic reimbursement disparities, revealing that procedures involving female anatomy are frequently reimbursed at lower rates than analogous surgeries performed on male anatomy [23]. These discrepancies, compounded by the lack of standardized training in advanced excisional techniques, place a disproportionate burden on patients. As a result, patients may experience limited access to experienced surgeons, delayed diagnoses, and increased out-of-pocket costs for high-quality care, ultimately perpetuating inequities in women’s health.

Strengths and limitations of the study

This study offers valuable insight into the relationship between surgical training and outcomes in endometriosis care, with its primary strength being the comparative analysis of surgical characteristics and postoperative outcomes across OBGYN subspecialties. This approach presents a novel perspective on how subspecialty training can influence clinical practice and patient experience. Although standardized pain scores were not available, physician documentation enabled the classification of postoperative pain into meaningful categories such as persistence or improvement. Some limitations were noted, including a relatively small sample size that may have contributed to overestimation of ORs, as well as missing data on pain assessment and the intraoperative diagnosis of DE. However, the low proportion of missing values for postoperative pain assessment supports the reliability of the findings. Moreover, objective surgical parameters, such as the surgical techniques used and the proportion of patients with DE who underwent excision of this form of endometriosis, offered valuable insights that supported the study’s aims. Overall, the findings contribute meaningfully to the growing evidence on the impact of surgical training in endometriosis management and underscore the need for future studies aimed at standardizing care and improving surgical education.

## Conclusions

Our data highlight significant variation in endometriosis surgical management across OBGYN subspecialties, with MIGS surgeons more likely to adopt minimally invasive and excisional techniques. However, excision of DE remained limited, even among MIGS. Persistent postoperative pain was common, though these findings should be interpreted cautiously given the small sample size and lack of standardized pain measurement. Overall, our results underscore the need for standardized surgical training and competency benchmarks to ensure consistent, high-quality care for endometriosis patients. Further research is essential to evaluate long-term outcomes, which will be critical for guiding future clinical practice and improving patient care.
